# A Giant Intracranial Hydatid Cyst in an Eleven-Year-Old Boy

**DOI:** 10.5334/jbr-btr.1211

**Published:** 2017-02-01

**Authors:** Taj Mohammad Waziri, Vikram Rao Bollineni, Riduan Kadi, Johan De Mey

**Affiliations:** 1 UZ Brussel, BE

**Keywords:** Intracranial hydatid disease, MRI spectroscopy, Cerebral cystic echinococcosis and MRI

An 11-year-old boy was assessed for complaints of visual disturbance and left temporal facial paralysis. His medical history and family history were unremarkable. Otological and neurological examinations indicated normal findings. Fundoscopic examination revealed papilledema. Noncontrast-computed tomography detected a voluminous, unilocular, thin-walled lesion without internal solid components in the right temporal lobe. This large cystic lesion had a density similar to that of cerebrospinal fluid (CSF) and showed no enhancement on contrast-enhanced computed tomography (Figure 1). The lesion caused the displacement of midline structures to the left side, left lateral ventricle compression, hydrocephalus, and subfalcine and transtentorial herniation (Figure 2). The cyst showed low signal intensity in T1-weighted images (T1WI) and high signal intensity in T2-weighed images (T2WI).

**Figure 1:**
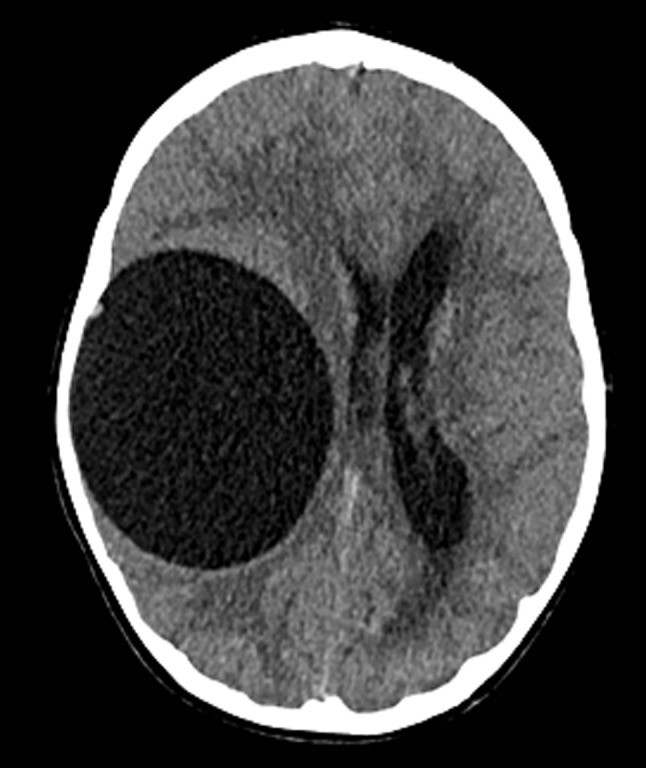
Axial image of Non-contrast-computed tomography.

**Figure 2:**
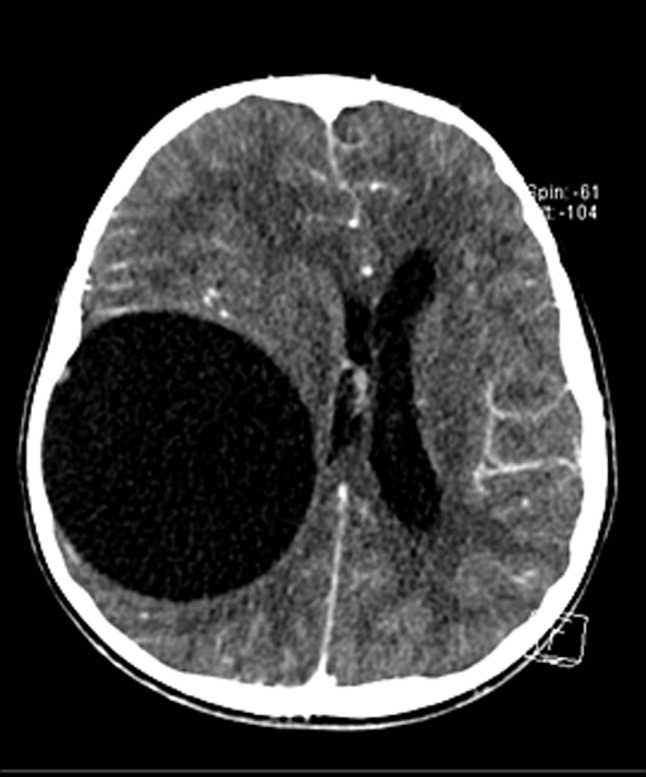
Axial image of Contrast-enhanced computed tomography.

In both T1WI and T2WI sequences (Figure 3), the cyst wall was observed as a hypointense ring without enhancement and diffusion restriction. T1WI revealed a unilocular cyst that was isointense to the CSF; T2WI showed a high signal intensity cyst with a well-defined hypointense rim. Histopathological findings correlated well with the radiological findings. Histological examination revealed a cyst surrounded by gliotic brain parenchyma, and the host tissue reaction indicated fibrocollagenous tissue lined by inflammatory cells and a few giant cells. Multiple calcific deposits and remnants of a hydatid cyst with characteristic laminated structures focally lined by a germinal layer were detected. The histological impression suggested alveolar echinococcosis.

**Figure 3:**
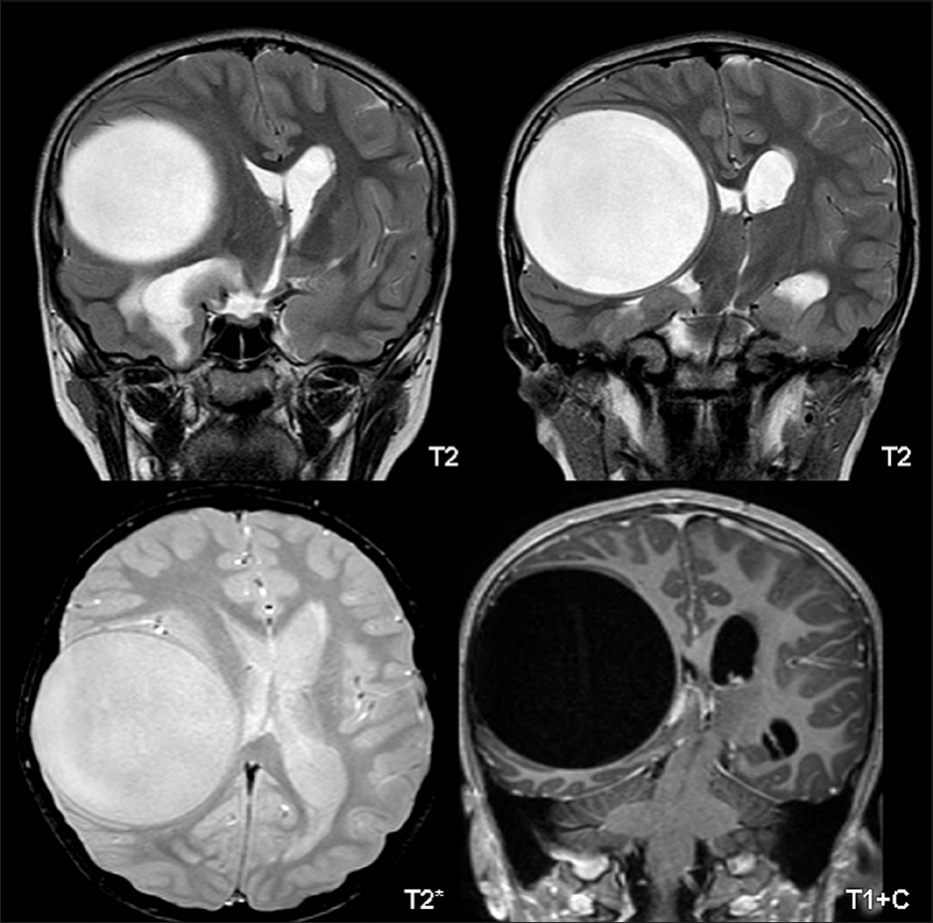
Axial and coronal images of MRI.

## Comment

Hydatid disease, also known as cystic echinococcosis, is a worldwide zoonosis caused by infection with the larval stage of the echinococcosis tapeworm. This parasite is incidentally ingested through contaminated food or beverages, and it first reaches the hepatic capillaries via the mesenteric and portal venous system, which explains why the liver is the primary site of echinococcal cysts. This disease is endemic to many places worldwide, particularly the Middle East, Australia, New Zealand, South America, and central and southern Europe. The liver and lungs are the most commonly involved organs.

Intracranial hydatid disease (IHD) is relatively rare and accounts for 1–2 percent of cases. IHD can occasionally be very difficult to diagnose on the basis of clinical and laboratory findings. Cerebral cystic echinococcosis lesions are usually singular, and multiple cerebral cysts are extremely rare. These cysts usually occur in the supratentorial area and involve the middle cerebral artery territory. IHD is a slow-growing benign lesion. Small cysts can be asymptomatic, but when enlarged, they can induce symptoms such as headache and vomiting; depending on the location of involvement; occasionally, ataxia, diplopia, hemiparesis, abducens nerve palsy, seizures, and coma can also occur.

Both CT and magnetic resonance imaging (MRI) are used to diagnose intracranial cysts [1]. On CT and MRI, a solitary cyst usually appears as a well-defined, low-attenuating, round lesion containing a smooth thin wall without wall enhancement postintravenous contrast injection. The cyst may cause extrinsic compression of the ventricular system, resulting in hydrocephalus and cerebral herniation. Differential diagnoses for hydatid cerebral cysts include arachnoid cysts, epidermoid cysts, enlarged perivascular spaces, neurological cysts, and porencephalic cysts.

Total surgical excision of the lesion is the treatment of choice in symptomatic patients. However, intraoperative rupture of the cyst is commonly encountered. Therefore, anthelminthic agents are usually administered for 2–4 weeks before surgery as an adjuvant treatment to prevent relapse.

## References

[1] Teke M, Göçmez C, Hamidi C (2015). Imaging features of cerebral and spinal cystic echinococcosis. Radiol Med.

